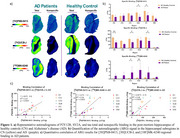# Investigating the Association Between P2Y12 Receptor, SV2A, and Tau Expression in Postmortem AD and Control Brains Using *In Vitro* Autoradiography

**DOI:** 10.1002/alz70856_102328

**Published:** 2025-12-25

**Authors:** Hannah Le, Roger Raymond, Neil Vasdev, Ruiqing Ni, Chao Zheng

**Affiliations:** ^1^ University of Toronto, Toronto, ON, Canada; ^2^ The Centre for Addiction and Mental Health, Toronto, ON, Canada; ^3^ ETH Zurich & University of Zurich, Zurich, Switzerland

## Abstract

**Background:**

Synaptic vesicle glycoprotein 2A (SV2A), a biomarker for synaptic density; purinergic receptor P2Y12R, a marker of microglia activation; and hyperphosphorylated tau have been extensively studied in Alzheimer's disease (AD), with growing interest in how these factors affect each other and enhance their impact. *In vitro* autoradiography (ARG) studies were carried out to evaluate the expression and distribution of P2Y12R, SV2A, and tau using [^3^H]PSB‐0413, [^3^H]UCB‐J and [^18^F]MK‐6240 respectively, in postmortem brain tissues from AD versus cognitively normal health controls (CN). Binding correlation analysis was also conducted.

**Method:**

[^18^F]MK‐6240 was synthesized in‐house, whereas [^3^H]PSB‐0413 and [^3^H]UCB‐J were custom synthesized (Novandi AB, Sweden). Thin section ARG binding assays were performed on post‐mortem hippocampal/BA28 and entorhinal cortex tissues from CN (*n* = 3) and AD patients (*n* = 5). Radiotracer binding was quantified using digital ARG and non‐specific binding was determined through homologous blockage with 10 µM of Suramin, Levetiracetam, and cold MK‐6240, known ligands for P2Y12R, SV2A and tau, respectively.

**Result:**

[^3^H]PSB‐0413 autoradiography revealed significant decreased binding in the subiculum (SUB) and entorhinal cortex (EC) of AD compared to CN. [^3^H]UCB‐J ARG showed significant decrease in the dentate gyrus (DG), CA1, and EC of AD compared to CN. [^18^F]MK‐6240 showed strong binding to tau aggregates throughout the hippocampus of AD, with minimal off‐target binding in CN, with a significant increased in binding observed in AD patients in CA1 and SUB compared to CN. When comparing binding in subregions in AD patients, [^3^H]UCB‐J (R^2^ = 0.57, *p* < 0.0001) and [^3^H]MK‐6240 (R^2^ = 0.57, *p* < 0.0001) have a significant association with [^3^H]PSB‐0413. The two tracers also share a significant correlation themselves (R^2^ = 0.48, *p* =  0.0002).

**Conclusion:**

This study demonstrates that SV2A levels decrease while tau accumulations increase in AD brains with [^3^H]UCB‐J and [^18^F]MK‐6420 respectively, and our data is consistent with *in vivo* human PET imaging studies of SV2A and tau. We also showed that P2Y12R is decreased in AD brains with [^3^H]PSB‐0413. Positive correlation between regional binding of the three tracers was shown in AD patients.